# Item Response Model Validation of the German ICD-11 International Trauma Questionnaire for PTSD and CPTSD

**DOI:** 10.32872/cpe.5501

**Published:** 2021-12-23

**Authors:** Daniel Christen, Clare Killikelly, Andreas Maercker, Mareike Augsburger

**Affiliations:** a Division of Psychopathology, Department of Psychology, University of Zurich, Zurich, Switzerland; Philipps-University of Marburg, Marburg, Germany

**Keywords:** International Trauma Questionnaire, ITQ, ICD-11, validation, PTSD, CPTSD, item response theory, German translation

## Abstract

**Background:**

In the 11th revision of the International Classification of Diseases (ICD-11) posttraumatic stress disorder (PTSD) and the complex variant (CPTSD) were newly conceptualised. The International Trauma Questionnaire (ITQ) was developed as a brief self-report measure to screen for both disorders. The English original version has been rigorously tested and presents convincing psychometric properties. The aim of the current study was to validate the German version by means of item response theory (IRT).

**Method:**

This is a secondary analysis of a representative, trauma-exposed adult sample from the German general population (N = 500). 1- and 2-parameter logistic IRT models (i.e. examination on an item level), diagnostic rates and confirmatory factor analyses were calculated.

**Results:**

All items showed good model fit and acceptable to good performance aligning with the items of the English original except for item C1 (Long time to calm down) which had a high endorsement rate and a low discriminatory power yielding low information gain. CPTSD diagnostic rate of 3.2% was lower than in comparable literature. Confirmatory factor analysis deemed the six first-order, two second-order factors model superior.

**Conclusion:**

Measurement and factorial validity of the German version of the ITQ was confirmed. The German translation matches the English original in most psychometric properties and can thus be used for research and clinical practice.

## Highlights

- The ITQ is a short self-report questionnaire measuring ICD-11 PTSD and CPTSD symptoms. There is a lack of evidence regarding the validity of the German translation.
- 1- and 2-parameter logistic Item Response Theory models were calculated and are comparable to the English original.
- Evidence for the measurement and factorial validity of the German translation was found.
- Confirmation of these results and further examination of the German ITQ are desirable.

## ICD-11 PTSD and CPTSD

In 2018, the World Health Organization (WHO) released the ICD-11 in which the two diagnoses posttraumatic stress disorder (PTSD) and the complex “sibling” diagnosis (CPTSD) were redefined and newly conceptualised (WHO, 2018). This reorganization aimed at improving clinical applicability and intercultural adaptation of the diagnoses for example by including a limited number of symptoms and clear delineation from other disorders (Keeley, Reed, Roberts, Evans, Medina-Mora, et al., 2016; Reed, 2010).

The diagnosis of PTSD consists of three symptom clusters (re-experiencing in the present, avoidance, and perception of current threat) in response to a traumatic event. Symptoms must persist for several weeks and cause significant impairment. Regarding CPTSD, three more symptom clusters called *Disturbances in Self-Organization* (DSO), must be clinically endorsed in addition to the presence of PTSD symptoms: problems in affect regulation, negative self-concept, and difficulties in relationships. The two diagnoses are mutually exclusive (WHO, 2018).

A growing body of evidence has confirmed the usefulness of these ICD-11 conceptualizations of PTSD and CPTSD. For instance, regarding the factorial structure of PTSD the three-factor structure has been demonstrated in various studies (e.g. Hansen, Hyland, Armour, Shevlin, & Elklit, 2015; Hyland, Brewin, & Maercker, 2017). For CPTSD two superordinate factors (PTSD and DSO) with six subordinate factors (symptom clusters) were the best-fitting models (Hyland, Shevlin, Elklit, et al., 2017; Nickerson et al., 2016; Shevlin et al., 2017). Several studies found clearly distinctive symptom profiles for individuals with PTSD and CPTSD by means of latent class and profile analyses (e.g. Knefel, Garvert, Cloitre, & Lueger-Schuster, 2015; Sachser, Keller, & Goldbeck, 2017). For CPTSD, divergent validity was found regarding Borderline Personality Disorder by means of latent class analyses (Cloitre, Garvert, Weiss, Carlson, & Bryant, 2014) and by means of a network analysis (Knefel, Tran, & Lueger-Schuster, 2016). A vignette-based study with international mental health experts found that the diagnostic guidelines for ICD-11 C/PTSD provide substantial clarifications in the diagnostic framework in comparison to ICD-10 (Keeley, Reed, Roberts, Evans, Robles, et al., 2016). Nevertheless, the ICD-11 concept for C/PTSD is not without controversies. For instance, in a network analysis with Israeli men, Gilbar (2020) found no clear boundaries between ICD-11 C/PTSD, depression, and anxiety symptoms. Finally, Møller, Augsburger, Elklit, Søgaard, and Simonsen (2020) compared measured ICD-11 C/PTSD and active ICD-10 diagnoses in Danish psychiatric outpatients and found an overlap between ICD-11 CPTSD and ICD-10 affective, personality, anxiety, behavioural, and emotional disorders.

## Development of the International Trauma Questionnaire

New diagnoses require accurate measuring instruments that are well conceptualized and validated. The International Trauma Questionnaire (ITQ) was developed to serve this purpose for ICD-11 PTSD and CPTSD (Cloitre, Roberts, Bisson, & Brewin, 2015; Cloitre et al., 2018). Items were developed in an iterative process based on criteria formulation from the *Diagnostic and Statistical Manual of Mental Disorders* (DSM) 5 PTSD (Brewin et al., 2009), the results of the DSM-IV field trials which assessed the most frequently reported CPTSD symptoms (van der Kolk et al., 2005), and a consensus survey among expert clinicians (Cloitre et al., 2011). The initial English ITQ resulted in a preliminary version with 28 items (Cloitre et al., 2015). Studies provided support for this version's factorial, discriminant, and convergent (Karatzias et al., 2016) as well as predictive validity (Hyland, Shevlin, Brewin, et al., 2017). In a last step, the number of items was reduced to 12 to conform to the organizing principle of ICD-11 that disorders should focus on a limited but central set of symptoms. This was done by assessing the psychometric properties of the items using item response theory (IRT) models (Cloitre et al., 2018).

In the validation study of the English ITQ, Cloitre et al. (2018) applied confirmatory factor analyses and IRT to data from both a community and a clinical sample with trauma exposure. For both, the PTSD and DSO cluster groups, a 1- and a 2-parameter logistic IRT model were compared. The 1-parameter model had a superior fit regarding all clusters except for the DSO items in the community sample. Differential item functioning was tested with multigroup IRT models comparing the two samples and showed adequacy of the ITQ for both of them. Rates of indicated diagnosis (diagnostic rates) of 5.3% PTSD and 12.9% CPTSD in the community sample and 14.6% PTSD and 61.1% CPTSD in the clinical sample were found mirroring outcomes from previous versions of the ITQ and pre-existing literature. Regarding the latent structure, Redican et al. (2021) conducted a systematic review of studies using factor analysis and mixture modelling. They found that the two-factor second-order model (the six symptom clusters as subordinate factors, PTSD and DSO cluster groups as superordinate factors) was consistently deemed the optimal model in clinical samples whereas in most community samples the six-factor model (the six symptom clusters each measured by two items) was preferred. Both models as well as the results of mixture modelling indicate that the ITQ can distinguish between PTSD and CPTSD. In summary the studies investigated by Redican et al. (2021) suggest that the ITQ is a valid measure of ICD-11 C/PTSD.

Up to now, the ITQ has been frequently applied (Cloitre et al., 2019; Hyland, Shevlin, Fyvie, Cloitre, & Karatzias, 2020; Karatzias et al., 2019; Redican et al., 2021) and recently examined the impact of COVID-19 (Tsur & Abu-Raiya, 2020). The ITQ has been translated into different languages and is publicly available (https://www.traumameasuresglobal.com/itq). The German translation was done by Lueger-Schuster, Knefel, and Maercker (2015/2018) but has not yet been validated.

## Aim of the Study

In order to be used in clinical practice and research regarding all related areas of C/PTSD the German translation of the ITQ needs to be clinically validated (Delahaye et al., 2015). So far, such a validation is still missing. Furthermore, additional investigations of the ITQ on the item level as well as data about the constructs of C/PTSD would support the understanding of these disorders and thus promote this field of research and its benefits. Therefore, this study aimed to validate the German translation of the ITQ by estimating 1- and 2-parameter logistic IRT models to examine item characteristics.

## Method

### Participants and Procedures

This is a secondary analysis of the data presented in Maercker, Hecker, Augsburger, and Kliem (2018). With the assistance of a scientific demographic consulting company (USUMA, Berlin, Germany) a representative sample for the German general population was selected. Participants were visited by a study assistant (trained layperson) and informed about the study. All participants or caregivers for minors provided written informed consent. Measurements were self-rating questionnaires except for the sociodemographic data. Overall, 2524 persons between the age of 14 and 99 years completed the assessment between January and March 2016. Since there is a version of the ITQ designed for children and adolescents from 7 to 17 years (Haselgruber, Sölva, & Lueger-Schuster, 2020), participants under the age of 18 (*n* = 84) were excluded. Further, participants with no traumatic event (*n* = 1774) and with missing values on all items of the ITQ (*n* = 166) were excluded. This resulted in a sample of *N* = 500 for the current study. Ethical approval for the study was granted (452-15-21122015, University of Leipzig, Medical School). Details are reported in Maercker et al. (2018). The mean age of participants was 52.41 years (*SD* 17.46, range 18-93).

### Measures

Only variables relevant for the current study are reported here. For further information, see Maercker et al. (2018).

#### Sociodemographic Data

Sex, age, family and partnership status, educational background, and employment status were assessed. The data is shown in Table 1.

**Table 1:** Sociodemographic Data

Variable / Category	Response	
	%	*n*
Sex		
Female	53.0	265
Male	47.0	235
Family status		
Married/living together	36.4	182
Married/living separated	3.4	17
Single	30.0	150
Divorced	16.2	81
Widowed	13.8	69
No answer	0.2	1
Living with a partner		
Yes	12.0	60
No	49.8	249
No answer	38.2	191
Educational background		
No or basic school leaving certificate	36.4	182
Intermediate school leaving certificate	37.2	186
Advanced school leaving certificate (university entrance level) or university degree	26.2	131
Other	0.2	1
Employment status		
Employed (full- or part-time)	47.8	239
Currently not working/unemployed	12.6	63
Studying	5.0	25
Retired	32.6	163
No answer	2.0	10

#### Traumatic Events

The trauma list of the Munich version of the Composite International Diagnostic Interview PTSD module (Perkonigg, Kessler, Storz, & Wittchen, 2000; Wittchen & Pfister, 1997) was applied. It assesses exposure to eight traumatic events (war, physical violence, rape, natural disaster, sexual abuse in the childhood, severe accident, kidnaping, life threatening illness) in addition to the category "other severe events and catastrophes" and witnessed events. “Other events” were counted if they met the definition of a traumatic event. Of the participants 14.6% (*n* = 73) reported having experienced war, 26.2% (*n* = 131) physical violence, 10.2% (*n* = 51) rape, 8.6% (*n* = 43) natural disaster, 9.8% (*n* = 49) sexual abuse in the childhood, 29.6% (*n* = 148) severe accident, 1.8% (*n* = 9) kidnapping, 18.0% (*n* = 90) life threatening illness, 41.6% (*n* = 208) witnessed an event and 3.8% (n = 19) other kinds of traumatic events. 59.2% (*n* = 296) of participants reported having experienced one traumatic event. 40.8% (*n* = 204) reported two or more traumatic events (mean number of experienced traumatic events = 1.66, *SD* = 1.01).

#### International Trauma Questionnaire (ITQ)

The German version of the ITQ was used (Lueger-Schuster et al., 2015/2018). This version has already been used in several studies, e.g. with survivors of institutional abuse (Lueger-Schuster et al., 2018) and in international network analyses (Knefel et al., 2019; Knefel et al., 2020). The ITQ assesses each of the three clusters of PTSD (P1-P6) and DSO (C1-C6) by two items as well as three additional items for functional impairment for PTSD (P7-P9) and DSO (C7-C9) each. Items are answered on a five-point Likert scale ranging from "0 = Not at all" to "4 = Extremely". A symptom cluster/the functional impairment is considered fulfilled if at least one of the items is clinically endorsed (score ≥ 2, “moderately”). A diagnosis of PTSD is indicated if every symptom cluster and the functional impairment item of the PTSD cluster group are fulfilled. If all symptom clusters and both functional impairments (across both PTSD and DSO cluster groups) are fulfilled, a diagnosis of CPTSD is indicated. In the current study the first item for functional impairment of the DSO cluster was not measured due to survey item restrictions.

### Statistical Analysis

Statistical analyses were conducted using the Software R (version 3.6.2) with the package *ltm* (Rizopoulos, 2018).

#### Data Preparation

Missing values in the ITQ (present in *n* = 38, max. of 5 missing values) was imputed by multiple (five) imputation. Analyses with imputed values were compared with complete cases. No significant differences were found.

#### Analysis of Dimensionality

To choose appropriate IRT models, an analysis of dimensionality of the symptom items for PTSD, DSO and both together (ITQ) was conducted (Mair, 2018). Dimensionality was explored with categorial principal component analyses, item factor analysis models and exploratory factor analyses as no assumption about the factor structure of the translation was made. However, since there are a lot of studies about the factor structure of the ITQ in other languages (Redican et al., 2021) a confirmatory factor analysis was done in addition. More detailed information is reported in Appendix A (see Supplementary Materials).

#### IRT Models

IRT focusses explicitly on which conclusions can be drawn from measured values/manifest variables (e.g. answer to an item) on underlying constructs/traits (θ) (e.g. PTSD) which are assumed to have a probabilistic relationship that can be modelled with different grades of complexity. One of the simplest models is the 1-parameter logistic model (Rasch, 1993). It models the dichotomous answer to an item in dependence of θ with a difficulty parameter which indicates at which level of θ the probability of endorsing that item is .5. The more complex model is the 2-parameter logistic model (Birnbaum, 1968) with an additional discrimination parameter, which indicates the discriminatory power of an item (Moosbrugger & Kelava, 2007). Using the marginal maximum likelihood method, unidimensional 1-parameter logistic and 2-parameter logistic IRT models were calculated for the PTSD and DSO cluster groups with dichotomized items. Model fit was assessed via the z-statistics to investigate whether item parameters were significantly different from zero (*z* > 1.65) and models were re-run with randomly generated data and compared to the real dataset. Here, a *p*-value < .05 indicated that an item did not fit the model. 1-parameter and 2-parameter model within each cluster were compared using the Akaike Information Criterion (AIC) (Akaike, 1974) and Bayesian Information Criterion (BIC) (Schwarz, 1978) with lower values indicating the better model. A difference in those values of ≥ 10 was considered "significant" (Raftery, 1995) and on the basis of parsimony, the 1-parameter model was chosen unless the criteria indicated the 2-parameter model is superior. Finally, item information curves were calculated to visualize item parameters and compare the information richness gained (Wood & Molenaar, 2017). Estimated item difficulty and discrimination parameters along with endorsement rates were compared to the results for the community sample of the analysis by Cloitre et al. (2018), as they used a similar method to validate the English version. Additionally, diagnostic rates for PTSD and CPTSD were compared to previous literature.

## Results

### Analysis of Dimensionality

Overall, the categorial principal component analysis as well as the criteria very simple structure and minimum average partial supported unidimensionality of PTSD and DSO cluster groups. Exploratory factor analysis models with different numbers of factors all showed insufficient fit and all criteria values of the item factor analysis models laid very close to each other. Confirmatory factor analysis found the six first-order, two second-order factors model to be superior. More detailed results of the analysis of dimensionality are reported in Appendix B (see Supplementary Materials).

### IRT Models

Model fit was good for all four estimated models (1-parameter logistic and 2-parameter logistic models for each the PTSD and DSO cluster groups): None of the *z*-statistic values were ≤ 1.65 and thus item parameters were significantly different from zero. Item fit within models yielded *p*-values of > .05 for all items, confirming their fit. AIC and BIC of the 1-parameter and 2-parameter models within each cluster group are shown in Table 2. For PTSD, no model was favoured according to the AIC (difference < 10) and according to the BIC the 1-parameter model was superior. For the DSO cluster group both criteria indicated the 2-parameter model was better.

**Table 2:** Comparison of the 1- and 2-Parameter Logistic Models

Model	AIC	BIC
PTSD		
1PL	2700.48	2729.98
2PL	2700.39	2750.97
DSO		
1PL	2169.03	2198.54
2PL	2114.77	2165.34

*Note*. AIC = Akaike information criterion; BIC = Bayesian information criterion; DSO = Disturbances in Self-Organization; PTSD = posttraumatic stress disorder; 1PL = 1-parameter logistic; 2PL = 2-parameter logistic.

**Figure 1:**
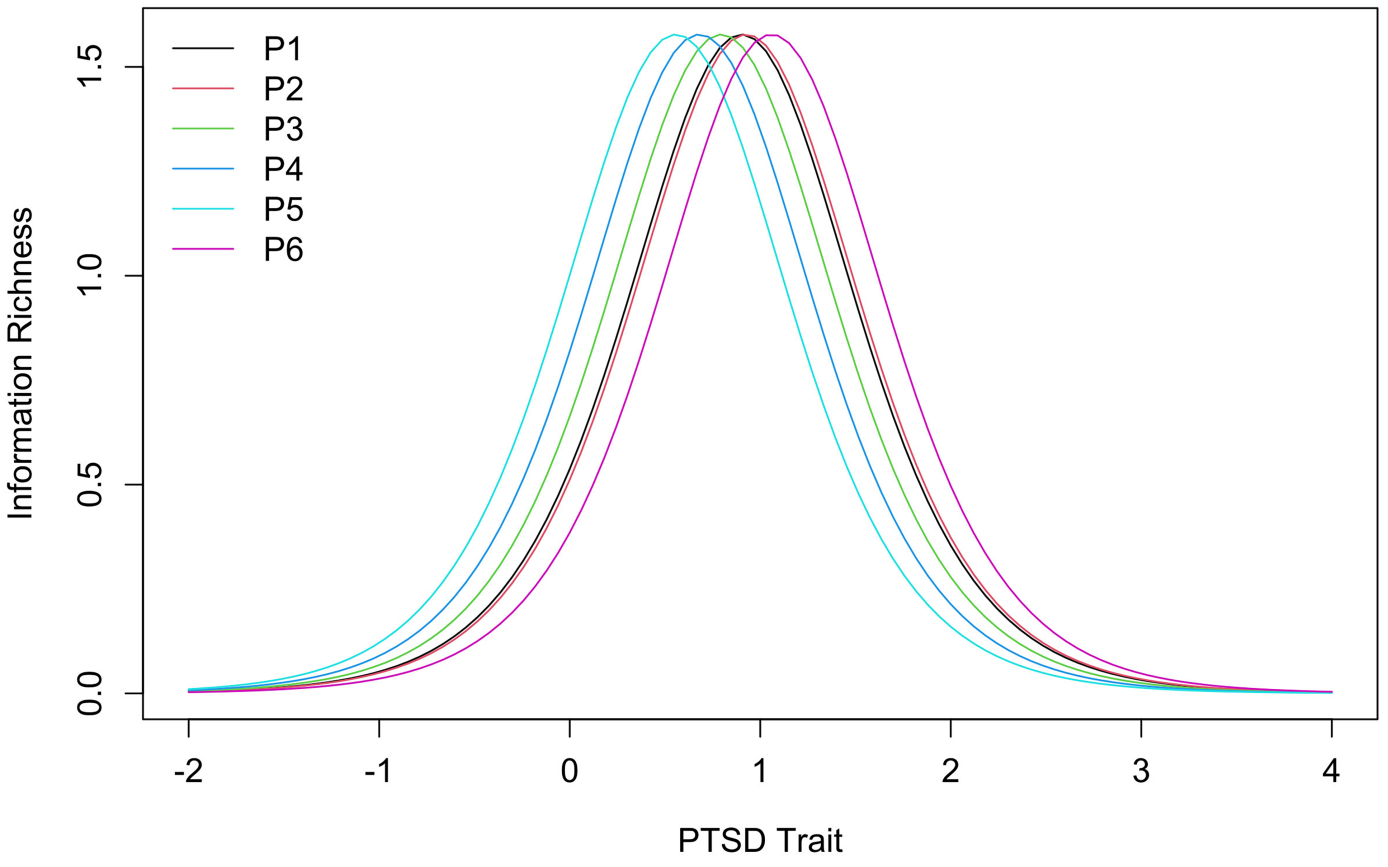
Item Information Curves of the 1-Parameter Logistic Model for the PTSD Cluster Group

Item information curves of the 1-parameter model for the PTSD cluster group are visualized in Figure 1. Item difficulty (left-right shift) showed a narrow, even distribution except for items P1 (Upsetting dreams) and P2 (Powerful images or memories) whose item information curves practically overlapped.

**Figure 2:**
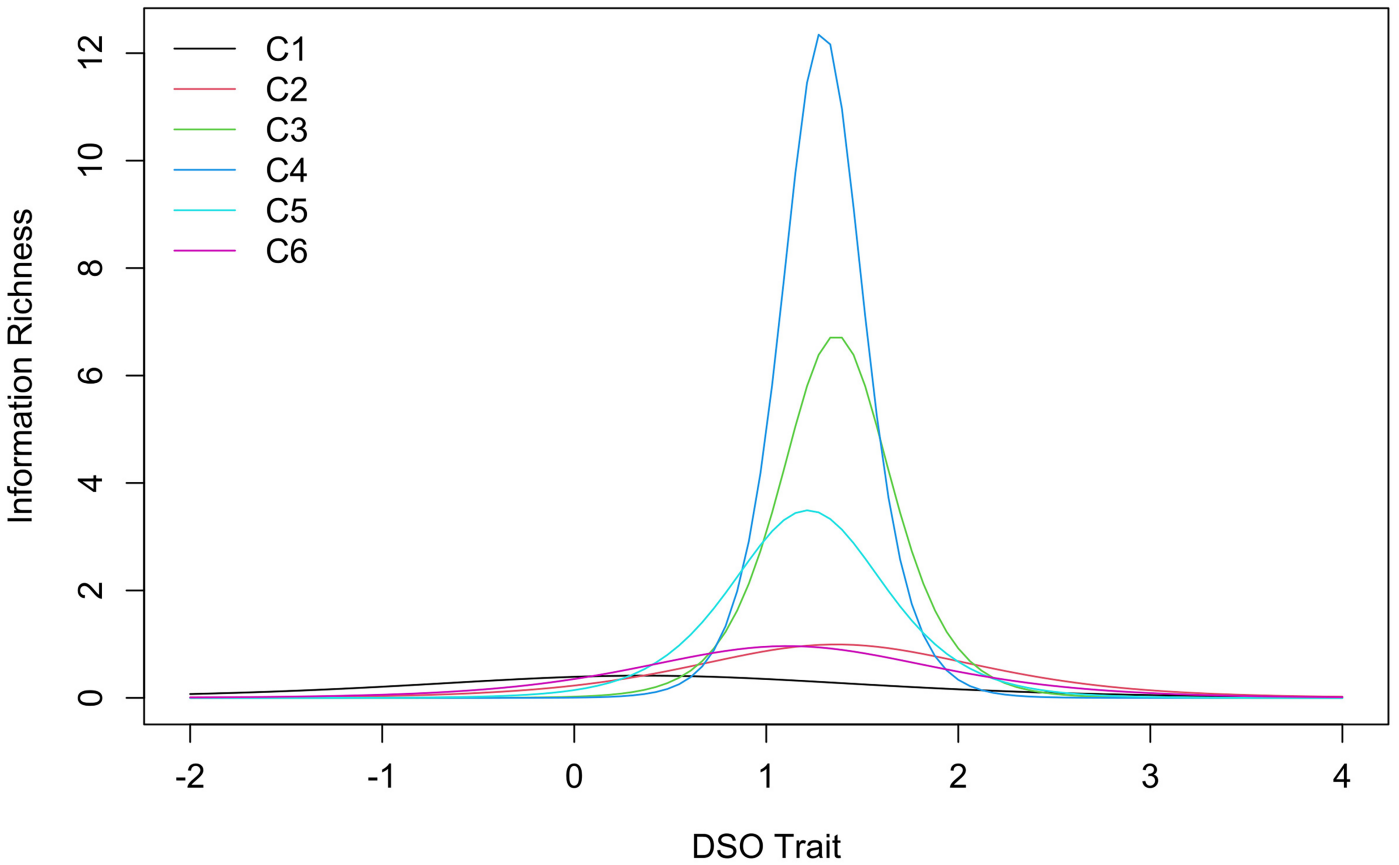
Item Information Curves of the 2-Parameter Logistic Model for the DSO Cluster Group

Item information curves of the 2-parameter model for the DSO cluster group are visualized in Figure 2. Item difficulty showed a narrow distribution as well, except for item C1 (Long time to calm down) whose item information curve was an outlier on the lower end of DSO trait. Discriminatory power varied largely with item C4 (I feel worthless) on the upper end with a tall narrow curve and item C1 on the lower end showing a flat wide curve.

Item parameters of the models and the endorsement rates from the current study as well as the community sample of the study by Cloitre et al. (2018) are shown in Table 3. For PTSD, endorsement rates of the two studies spread over similarly sized ranges. The ranges overlapped with the highest two endorsement rates of the present study within the range of the study by Cloitre et al. (2018) and their three lowest rates within the range of the present study. Discrimination parameters were the same for all items of the German ITQ and for the items of each cluster in the English ITQ which is due to the use of slightly different 1-parameter logistic models. Discrimination parameters of the German version were lower. In contrast, item difficulty parameters of this study were higher than those reported by Cloitre et al. (2018). Still, difficulty parameters within the two studies scattered over similarly sized areas. Item information curves of the non-favoured models are reported in Appendix C (Supplementary Materials).

**Table 3:** Endorsement Rates and Item Parameters of the Present Study and Cloitre et al. (2018)

Item	Endorsement (%)		Discrimination (*SE*)		Difficulty (*SE*)	
	Present study	Cloitre et al.	Present study	Cloitre et al.	Present study	Cloitre et al.
PTSD						
P1	22.8	26.8	2.50 (0.17)	3.89 (0.17)	0.91 (0.08)	0.67 (0.05)
P2	22.0	31.8	2.50 (0.17)	3.89 (0.17)	0.94 (0.08)	0.51 (0.04)
P3	25.9	37.7	2.50 (0.17)	6.32 (0.58)	0.79 (0.08)	0.32 (0.04)
P4	28.9	34.6	2.50 (0.17)	6.32 (0.58)	0.69 (0.07)	0.40 (0.04)
P5	32.5	36.0	2.50 (0.17)	6.53 (0.62)	0.56 (0.07)	0.36 (0.04)
P6	19.2	29.5	2.50 (0.17)	6.53 (0.62)	1.06 (0.08)	0.55 (0.04)
DSO						
C1	40.9	42.8	1.34 (0.21)	2.78 (0.21)	0.36 (0.10)	0.22 (0.05)
C2	15.2	36.1	2.09 (0.32)	3.79 (0.33)	1.32 (0.13)	0.41 (0.04)
C3	9.8	36.3	5.48 (1.32)	6.64 (0.91)	1.34 (0.08)	0.37 (0.04)
C4	11.0	34.5	7.05 (2.22)	8.41 (1.43)	1.27 (0.07)	0.42 (0.04)
C5	13.4	40.3	3.64 (0.61)	5.69 (0.74)	1.21 (0.09)	0.27 (0.04)
C6	20.2	39.6	2.03 (0.29)	4.54 (0.48)	1.08 (0.11)	0.30 (0.04)

*Note*. C1 = Long time to calm down; C2 = Feeling numb; C3 = Feeling like a failure; C4 = Feeling worthless; C5 = Feeling cut off from people; C6 = Finding it hard to stay emotionally close to people; DSO = Disturbances in Self-Organization; PTSD = posttraumatic stress disorder; P1 = Upsetting dreams; P2 = Powerful images or memories; P3 = Avoiding internal reminders; P4 = Avoiding external reminders; P5 = Being “super-alert”; P6 = Feeling jumpy; *SE* = standard error.

For DSO, endorsement rates found in this study were generally lower than the ones reported by Cloitre et al. (2018). Endorsement rate of item C1 (Long time to calm down) constitutes an anomaly within the DSO cluster group of the German version as it is more than double the size of the next highest endorsement rate. This was not the case for any other endorsement rate including the same item in the English version. Discrimination parameters of the German version were descriptively lower and difficulty parameters higher. In the German version the parameters of item C1 again did not align with the other items. 5.0% (*n* = 25) in the current sample exceeded the threshold for an indication of PTSD diagnosis and 3.2% (*n* = 16) indicated CPTSD diagnosis.

## Discussion

This study aimed to validate the German translation of the ITQ. This is essential for the scale to be clinically valid (Delahaye et al., 2015) and enhances the understanding of the C/PTSD disorder structure. Previously this was done successfully for the English version by Cloitre et al. (2018) with a similar analysis.

Due to the strong empirical support, unidimensional IRT models were calculated. For PTSD, the 1-parameter and for DSO, the 2-parameter logistic models were deemed superior. This suggests that the items of the PTSD cluster group do not vary enough in discriminatory power for having to consider this parameter in modelling, whereas in the DSO cluster group discriminatory power seems to vary too much to be omitted as a parameter.

PTSD items showed no excessive high or low endorsement rates and no outlier. Cloitre et al. (2018) found similar values but with higher overall average. This difference could be due to the translation however endorsement rates of all PTSD items were lower indicating that sample differences seem more likely. Such a difference could arise from the number of traumatic experiences in each sample since this is associated with higher probability of C/PTSD (Karatzias et al., 2016; Kolassa, Kolassa, Ertl, Papassotiropoulos, & De Quervain, 2010). In the community sample of Cloitre et al. (2018) the average number of traumatic experiences was 3.36 while in the current study it was 1.66 with the majority (59.2%) having experienced just a single traumatic event. This could explain higher PTSD traits and thus higher endorsement rates in the sample of Cloitre et al. (2018). Within the DSO cluster group item C1 (Long time to calm down) was an outlier in terms of endorsement rate and had the lowest discrimination parameter. This resulted in relatively little information gained from this item. In the English version, item C1 had a similar endorsement rate however it did not constitute an outlier and had a higher discrimination parameter. A possible reason for this difference could lie in the broader meaning of the German translation. While *upset* ("When I am upset, it takes me a long time to calm down.") represents feelings of worry, unhappiness, or anger, the German equivalent word *aufgeregt* additionally represents the feeling of pleasant anticipation as well as a physiological arousal or agitation. This could lead to higher endorsement of this item in the German translation.

All other DSO items had quite low endorsement rates compared to the PTSD cluster group as well as to the results of Cloitre et al. (2018). Since low endorsement rates were consistent over five items sample differences in CPTSD trait seem a more probable reason than the translation process itself. Differences in the average number in traumatic events could explain these different rates. To better understand the performance of item C1 and the low endorsement rates of items C2 to C6 it would be desirable to have future studies investigate this, for example, using polytomous IRT modelling with a community and a clinical sample.

Combined diagnostic rate of PTSD (5.0%) and CPTSD (3.2%) was 8.2% and thus lower than in comparable studies like Ben-Ezra et al. (2018) (9% PTSD and 2.6% CPTSD), Knefel et al. (2019) (12.9% PTSD and 20.6% CPTSD), and Hyland et al. (2020) (5.0% PTSD and 7.7% CPTSD). In the latter study with a nationally representative sample form Ireland the difference in diagnostic rates was mainly due to the difference in CPTSD rate. The CPTSD rate in the current study was most probably underestimated by omission of item C7 (concern about social life) due to survey item restrictions. Diagnostic rates can thus be considered in accordance with pre-existing literature.

The prevalence ratio of PTSD and CPTSD in different samples is subject to an ongoing scientific debate (Cloitre et al., 2018). There is a dominant hypothesis that community rates of PTSD should be higher than CPTSD while the reverse is true for trauma clinics (Brewin et al., 2009). However, this view is challenged by studies showing that multiple traumatic experiences are associated with higher rates of CPTSD than PTSD (Elklit & Shevlin, 2007) and that in community samples multiple experiences of traumatisation is more common than a single experience (Scott et al., 2013). In the present study the PTSD rate was higher than CPTSD. This is in line with the proposed hypothesis that in a community sample PTSD is higher. On the other hand, it contradicts the findings that multiple experiences of traumatisation may be more likely in community samples. In comparison to the diagnostic rates of Cloitre et al. (2018) (5.3% PTSD and 12.9% CPTSD) the 5.0% PTSD rate in the present study was similar whereas the 3.2% CPTSD rate was lower. Again, this difference is likely due to the omission of item C7 as well as a higher mean number of traumatic experiences in the sample of Cloitre et al. (2018).

Confirmatory factor analysis showed the six-factors and the six first-order, two second-order factors model to be of good fit and the latter to be superior. This coincides with other studies on community samples e.g. with the Italian (Somma, Maffei, Borroni, Gialdi, & Fossati, 2019) or the Korean translation (Choi, Kim, & Lee, 2021) and speaks for the validity of the German translation of the ITQ (Redican et al., 2021).

Using IRT methods, the German translation of the ITQ was investigated with a special focus on the item level. The results support the validity of all items except for item C1 (Long time to calm down). Additionally, confirmatory factor analysis too, pointed to the validity of the investigated questionnaire. Since the present study used data of a community sample the ITQ could not be tested for differential item functioning, i.e. different performance in a community than in a clinical sample. Differential item functioning, potential changes in diagnostic rates and applicability in clinical samples would be interesting to investigate in the future. Finally, this validation approach should be complemented with classical test theory (e.g., investigating convergent and divergent validity) in other studies.

### Limitations

Almost half of the individuals contacted refused to participate in the study. Although this is common in surveys of this kind, a potential selection bias cannot be excluded (Maercker et al., 2018). Individuals who experienced strong avoidance symptoms might have been overlooked due to the inclusion criteria of a subjectively most burdensome event. Further, not all CPTSD impairment items could be included in the study due to survey item restrictions. This did not impact IRT model estimation but might have influenced CPTSD diagnostic rate.

### Conclusion

The German translation of the ITQ can be considered a valid measure for ICD-11 C/PTSD. An exceptional case was the item C1 ("When I am upset, it takes me a long time to calm down."), which showed mismatched item parameters in comparison to other items of the same cluster group. As this is the first study to specifically examine the validity of the German version of the ITQ its findings are important in many regards. Having a validated measurement for ICD-11 C/PTSD in German supports future research and its benefits concerning the German speaking population worldwide as well as global research by providing data from this population. Research and benefits for the population apply to all related areas of C/PTSD, from the disorders themselves, over disorders specifically associated with stress, to anything including C/PTSD as a precursory, accompanying or resulting condition and be it about prevalence, prevention, intervention, rehabilitation, or others. Some studies already used the German translation of the ITQ prior to this validation (Knefel et al., 2019; Knefel et al., 2020; Lueger-Schuster et al., 2018). Results gained this way receive backup through the validation of the instrument. Besides research this study provides an important contribution to the clinical applicability of the ITQ and thus the health care of the German speaking population. As a validated instrument it can be used in practice to screen for ICD-11 C/PTSD, support the diagnostic process, accompany a (therapeutic) process and more. Further, the information gained about the performance of the translated items also furthers the understanding of the appropriate wording and combination of items to measure the C/PTSD constructs in German as well as opens the possibility to improve the ITQ. For the German ITQ, consider renaming the previous translation of "upset", e.g., using the German verb "aufgewühlt" or "aufgebracht". Future studies should try to confirm the present findings including a clinical sample to test for differential item functioning and changes in diagnostic rates and include item C7. Also of interest would be the investigation of different kinds of validity of the German ITQ to consolidate the findings here.

## Supplementary Materials

The Supplementary Materials contain the detailed method and results of the analysis of dimensionality and item information curves of the non-favoured IRT-models (for access see Index of Supplementary Materials below).

10.23668/psycharchives.5253 Supplement 1 Supplementary materials to "Item response model validation of the German ICD-11 International Trauma Questionnairefor PTSD and CPTSD"

ChristenD.
KillikellyC.
MaerckerA.
AugsburgerM.
 (2021). Supplementary materials to "Item response model validation of the German ICD-11 International Trauma Questionnaire for PTSD and CPTSD"
[Additional information]. PsychOpen. 10.23668/psycharchives.5253
PMC966722536398291

## References

[sp1_r1] Christen D. Killikelly C. Maercker A. Augsburger M. (2021). Supplementary materials to "Item response model validation of the German ICD-11 International Trauma Questionnaire for PTSD and CPTSD" [Additional information]. PsychOpen. 10.23668/psycharchives.5253 PMC9667225 36398291

