# Supplementary material for: Item Response Model Validation of the German ICD-11 International Trauma Questionnaire for PTSD and CPTSD
Source: Clin Psychol Eur. 2021 Dec 23;3(4):e5501. doi: 10.32872/cpe.5501 (PMC9667225; doi:10.32872/cpe.5501)
Supplement: Supplement 1 [file cpe-03-5501-s01.pdf]

Item Response Model Validation of the German ICD-11 International Trauma Questionnaire for PTSD and CPTSD

Appendices

Published in Clinical Psychology in Europe: https://doi.org/10.32872/cpe.5501

Daniel Christen[a]*, Clare Killikelly[a] https://orcid.org/0000-0003-2661-4521, Andreas Maercker[a] https://orcid.org/0000-0001-6925-3266, Mareike Augsburger[a] https://orcid.org/0000-0002-6564-0717

[a] Division of Psychopathology, Department of Psychology, University of Zurich, Zurich, Switzerland.

Corresponding Author: Daniel Christen, Binzmuehlestrasse 14, CH-8050 Zurich, Switzerland. Tel.: +41 79 572 88 01. E-mail: dc_christen@gmx.ch

Appendix A Method of Analysis of Dimensionality

To choose appropriate IRT models, an analysis of dimensionality of the symptom items for PTSD, DSO and both together (ITQ) was conducted (Mair, 2018). The dimensionality was analysed in several ways first with a categorial principal component analysis in which two components were extracted. Grouping of the loading of items was examined in loadings plots. Second, an exploratory factor analysis with tetrachoric correlations was applied. In scree tests with parallel analysis (Horn, 1965) the elbow (Cattell, 1966) and Kaiser criterion (Kaiser, 1960) and the comparison with resampled data was applied to assess the number of underlying factors. Additionally, the criteria very simple structure (Revelle & Rocklin, 1979), minimum average partial (Velicer, 1976), BIC (Schwarz, 1978) and sample size adjusted BIC (saBIC) (Sclove, 1987) were used. Higher values in very simple structure indicated a better fit, whereas the reverse was true for minimum average partial, BIC, and saBIC. Due to gathered information about the possible dimensionality up to this point exploratory factor analysis models with one, two and in the case of the ITQ seven factors but no assignment of items to the factors were calculated and compared via the root mean squared error of approximation (MacCallum, 2009) and the Tucker-Lewis Index (Tucker & Lewis, 1973). Root mean squared error of approximation values < .05 were considered as good model fit, .06-.08 as moderate and > .08 as poor (Browne & Cudeck, 1992). Tucker-Lewis Index values > 0.9 were considered good (Hu & Bentler, 1999). Third, item factor analysis models with one, two and three factors were calculated and compared with the AIC (Akaike, 1974), AIC correction for small sample size (Hurvich & Tsai, 1989), BIC and saBIC. Lower values indicated a better fit. Fourth and final, confirmatory factor analysis was done testing a a) one-factor b) two-factors c) six-factors d) two first-order one second-order factors e) six first-order one second-order factors and f) six first-order two second-order factors model. Model rating and comparison was done with the comparative fit index (Bentler, 1990), Tucker-Lewis Index, AIC, BIC, saBIC, root mean squared error of approximation and standardized root mean square residual (Hu & Bentler, 1999). For the comparative fit index values ≥ .95 and for the standardized root mean square residual values ≤ .08 were considered good model fit. For the other indices the same cut-offs as above were used.

Appendix B Results of Analysis of Dimensionality

Loadings plots of the categorial principal component analysis showed the loadings of the items of PTSD and DSO grouping closely together regarding one of the two extracted components. Parallel analyses (Horn, 1965) as well as the elbow (Cattell, 1966) and the Kaiser (Kaiser, 1960) criterion in the scree test of the exploratory factor analysis indicated one factor for PTSD and DSO cluster groups separately and two factors for the ITQ. The number of factors best fitting the exploratory factor analysis by very simple structure (Revelle & Rocklin, 1979), minimum average partial (Velicer, 1976), BIC (Schwarz, 1978) and saBIC (Sclove, 1987) are shown in Table B1.

Table B1. Number of Factors for Exploratory Factor Analysis Indicated by Various Criteria

	Number of factors		
Criterion	PTSD	DSO	ITQ
VSS, complexity 1	1	1	1
VSS, complexity 2	2	2	2
MAP	1	1	2
BIC	2	2	7
saBIC	2	2	7

Note. BIC = Bayesian information criterion. DSO = Disturbances in Self-Organization. ITQ = International Trauma Questionnaire. MAP = minimum average partial. PTSD = posttraumatic stress disorder. saBIC = sample size adjusted Bayesian information criterion. VSS = very simple structure.

Tucker-Lewis Index (Tucker & Lewis, 1973) was not good (> 0.9) for any calculated exploratory factor analysis model and root mean squared error of approximation (MacCallum, 2009) was poor (> .08) for all of them. AIC (Akaike, 1974), AIC correction for small sample size (Hurvich & Tsai, 1989), BIC and saBIC were lowest for the item factor analysis models with three factors in all cases except for BIC in DSO cluster group which was lowest for one factor. Values within a cluster group were all very similar with a maximal difference of < 4.8%. The fit indices of the models tested with confirmatory factor analysis are shown in Table B2.

Table B2. Fit Indices of the Models Tested With Confirmatory Factor Analysis.

Model	CFI	TLI	AIC	BIC	saBIC	RMSEA	SRMR
One-factor	0.67	0.60	4432.42	4533.57	4457.39	0.16	0.10
Two-factors	0.87	0.83	3999.30	4104.66	4025.31	0.11	0.07
Six-factors	0.97	0.95	3789.76	3954.13	3830.34	0.06	0.03
Two first-order, one second-order factors	0.87	0.83	3999.30	4104.66	4025.31	0.11	0.07
Six first-order, one second-order factors	0.91	0.87	3915.44	4041.88	3946.66	0.09	0.07
Six first-order, two second-order factors	0.97	0.95	3782.51	3913.16	3814.76	0.06	0.04

Note. AIC = Akaike information criterion. BIC = Bayesian information criterion. CFI = comparative fit index. RMSEA = root mean squared error of approximation. saBIC = sample size adjusted Bayesian information criterion. SRMR = standardized root mean square residual. TLI = Tucker-Lewis Index.

Comparative fit index (Bentler, 1990) and Tucker-Lewis Index was good (≥ .95 and > 0.9) for the six-factors and the six first-order, two second-order factors model. The same models were deemed moderate (.06-.08) by the root mean squared error of approximation while the other models were considered poor. AIC, BIC and saBIC all were lowest for the six first-order, two second-order

factors model. Standardized root mean square residual (Hu & Bentler, 1999) was good for all models except the one-factor model.

Appendix C Item Information Curves of the Non-Favoured Models

Item information curves of the 2-parameter logistic model for the PTSD cluster group are visualized in Figure C1. Item difficulty showed a narrow distribution with P1 (Upsetting dreams) and P2 (Powerful images or memories) peaking almost at the same level of θ. Discriminatory power varied although less than in the DSO 2-parameter model.

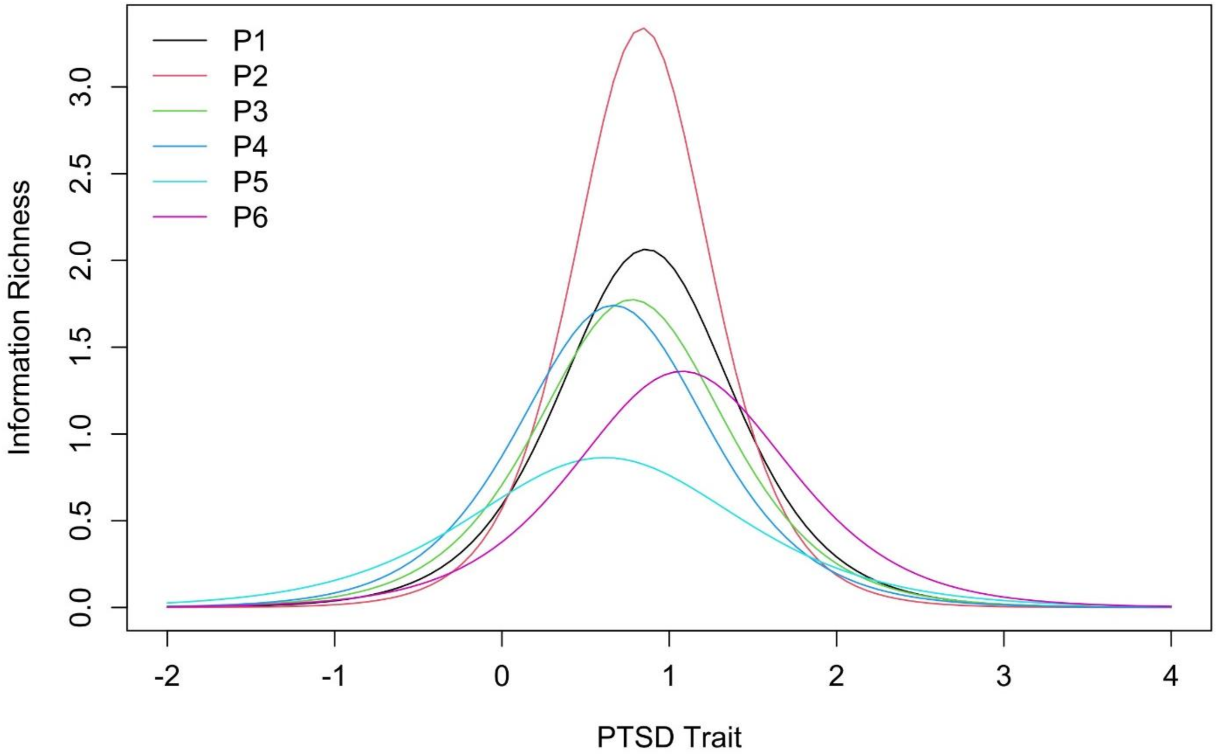

Figure C1. Item information curves of the 2-parameter logistic model for the PTSD cluster group.

Item information curves of the 1-parameter logistic model for the DSO cluster group are visualized in Figure C2. Item difficulty of the DSO 1-parameter model showed a narrow distribution except for C1 (Long time to calm down) whose item information curve constitutes an outlier towards lower DSO trait.

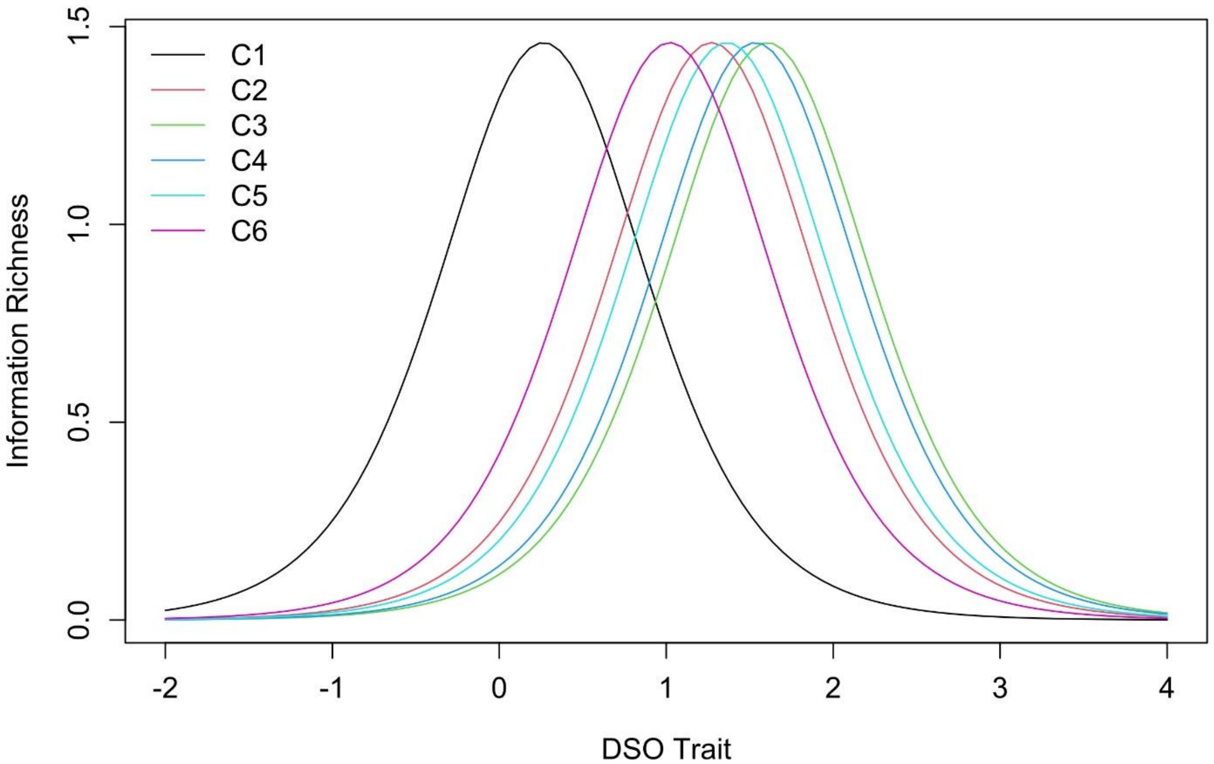

Figure C2. Item information curves of the 1-parameter logistic model for the DSO cluster group.

References

Akaike, H. (1974). A new look at the statistical model identification. IEEE Transactions on Automatic Control, 19(6), 716–723. doi:10.1109/TAC.1974.1100705Bentler, P. M. (1990). Comparative fit indexes in structural models. Psychological Bulletin, 107(2), 238–246. doi:10.1037/0033-2909.107.2.238

Browne, M. W., & Cudeck., R. (1992). Alternative ways of assessing model fit. Sociological Methods & Research, 21(2), 230–258. doi:10.1177/0049124192021002005

Cattell, R. B. (1966). The scree test for the number of factors. Multivariate Behavioral Research, 1(2), 245–76. doi:10.1207/s15327906mbr0102_10

Horn, J. L. (1965). A rationale and test for the number of factors in factor analysis. Psychometrika, 30(2), 179–185. doi:10.1007/BF02289447

Hu, L., & Bentler, P. M. (1999). Cutoff criteria for fit indexes in covariance structure analysis: Conventional criteria versus new alternatives. Structural Equation Modeling, A Multidisciplinary Journal, 6(1), 1–55. doi:10.1080/10705519909540118

Hurvich, C. M., & Tsai, C. L. (1989). Regression and time series model selection in small samples. Biometrika, 76(2), 297–307. doi:10.1093/biomet/76.2.297

Kaiser, H. F. (1960). The application of electronic computers to factor analysis. Educational and Psychological Measurement, 20(1), 141–151. doi:10.1177/001316446002000116

MacCallum, R. C. (2009). Factor analysis. In R. E. Millsap & A. Maydeu-Olivares (Eds.), The SAGE Handbook of Quantitative Methods in Psychology (pp. 123–147). London: SAGE Publications Ltd.

Mair, P. (2018). Modern psychometrics with R. Cham: Springer Nature Switzerland AG.

Revelle, W., & Rocklin, T. (1979). Very simple structure, an alternative procedure for estimating the optimal number of interpretable factors. Multivariate Behavioral Research, 14(4), 403–414. doi:10.1207/s15327906mbr1404_2

Schwarz, G. (1978). Estimating the dimension of a model. The Annals of Statistics, 6(2), 461–464. doi:10.1214/aos/1176344136

Sclove, S. L. (1987). Application of model-selection criteria to some problems in multivariate analysis. Psychometrika, 52(3), 333–343. doi:10.1007/BF02294360

Tucker, L. R., & Lewis, C. (1973). A reliability coefficient for maximum likelihood factor analysis. Psychometrika, 38(1), 1–10. doi:10.1007/BF02291170

Velicer, W. F. (1976). Determining the number of components from the matrix of partial correlations. Psychometrika, 41(3), 321–327. doi:10.1007/BF02293557
